# Spectral Method in Epidemic Time Series: Application to COVID-19 Pandemic

**DOI:** 10.3390/biology11121825

**Published:** 2022-12-14

**Authors:** Jacques Demongeot, Pierre Magal

**Affiliations:** 1 Université Grenoble Alpes, AGEIS EA7407, F-38700 La Tronche, France; 2 Université Bordeaux, IMB, UMR 5251, F-33400 Talence, France; 3 CNRS, IMB, UMR 5251, F-33400 Talence, France

**Keywords:** epidemic models, time series, spectral method, spectral truncation method, phenomenological models

## Abstract

**Simple Summary:**

This article aims to study the times series provided by data of the daily number of reported cases of COVID-19. During the COVID-19 pandemic, most people viewed the oscillations around the exponential growth at the beginning of an epidemic wave as the default in reporting the data. The residual is probably partly due to the reporting data process (random noise). Nevertheless, a significant remaining part of such oscillations could be connected to the infection dynamic at the level of a single average patient. Eventually, the central question we try to address here is: Is there some hidden information in the signal around the exponential tendency for COVID-19 data?

**Abstract:**

Background: The age of infection plays an important role in assessing an individual’s daily level of contagiousness, quantified by the daily reproduction number. Then, we derive an autoregressive moving average model from a daily discrete-time epidemic model based on a difference equation involving the age of infection. Novelty: The article’s main idea is to use a part of the spectrum associated with this difference equation to describe the data and the model. Results: We present some results of the parameters’ identification of the model when all the eigenvalues are known. This method was applied to Japan’s third epidemic wave of COVID-19 fails to preserve the positivity of daily reproduction. This problem forced us to develop an original truncated spectral method applied to Japanese data. We start by considering ten days and extend our analysis to one month. Conclusion: We can identify the shape for a daily reproduction numbers curve throughout the contagion period using only a few eigenvalues to fit the data.

## 1. Introduction

Modeling an epidemic peak requires precise knowledge of the daily data corresponding to new cases. One of the aims of the paper is to extract the value of the average daily reproduction numbers. The daily reproduction numbers vary from individual to individual and from day to day during the period of contagiousness of an individual. These numbers depend on the age of infection, i.e., the number of days since the individual contracted the infectious disease.

From a discrete model of the evolution of new daily cases, we propose to evaluate the average number $R_{0}\left(d\right)$ of secondary infected individuals produced by a single infected individual on each day *d* since his infection. For this purpose, on the top of the dominant eigenvalue, we will estimate from the data other significant subdominant eigenvalues (complex), which explain the modulation of the growth and allow better adequacy of the model to the data.

For that purpose, we reconsider the discrete-time epidemic model with the age of infection presented in Demongeot et al. [1]. This model is a discrete-time version of the Volterra integral formulation of the Kermack–McKendrick model with age of infection [2]. The variation of the number of susceptible individuals S(t) is given each day $t=t_{0},t_{0}+1,\ldots ,$ by

(1) $$S\left(t\right)=S_{0}-\sum_{d=t_{0}}^{t-1}N\left(d\right),\forall t\geq t_{0},$$

where S(t) is the number of susceptible individuals at time *t*, and N(t) is the daily number of new infected at time *t*. Throughout the paper, we use the following convention for the sum

$$\sum_{d=k}^{m}=0,\;\mathrm{whenever}\;m<k.$$

As a consequence, when $t=t_{0}$, (1) gives

$$S\left(t_{0}\right)=S_{0}.$$

We assume for simplicity that the epidemic starts from a single cohort of infected at time $t_{0}$, then the number of infectious individuals is given by

(2) $$I\left(t\right)=\left[\Gamma \left(t-t_{0}\right)I_{0}+\sum_{d=1}^{t-t_{0}}\Gamma \left(d\right)N\left(t-d\right)\right],$$

where $I_{0}$ is the number of infected individuals at time $t_{0}$, and Γ(d) is the probability for an infected to be infectious after *d* day of infection. In particular, we have Γ(0)=0.

We assume that the number N(t) of new infected at time *t* is the product of the transmission rate τ(t) with the number S(t) of susceptible individuals and the number I(t) of infectious at time *t*. That is,

(3) N(t)=τ(t)S(t)I(t).

By replacing I(t) by the right hand side of (2) in (3), we obtain

(4) $$N\left(t\right)=\tau \left(t\right)S\left(t\right)\left[\Gamma \left(t-t_{0}\right)I_{0}+\sum_{d=1}^{t-t_{0}}\Gamma \left(d\right)N\left(t-d\right)\right].$$

Now assuming that $\tau \left(t\right)=\tau_{0}$ and $S\left(t\right)=S_{0}$ are constant (over a short period of time), then we define the **daily reproduction numbers** as

$$R_{0}\left(d\right)=\tau_{0}\;S_{0}\;\Gamma \left(d\right),\forall d\geq 0.$$

The quantity $R_{0}\left(d\right)$ is the average number of secondary infected produced by a single infected on the day *d* since infection (see [1] for more details). Therefore, the **basic reproduction number** is the following quantity

(5) $$R_{0}=\sum_{d=1}^{n}R_{0}\left(d\right),$$

where *n* is the maximal duration (in days) of the infection.

Moreover, when $\tau \left(t\right)=\tau_{0}$ and $S\left(t\right)=S_{0}$ are constant, Equation (4) becomes a linear discrete time Volterra integral equation

(6) $$N\left(t\right)=\underset{\left(I\right)}{\underset{︸}{R_{0}\left(t-t_{0}\right)I_{0}}}+\underset{\left(\mathrm{II}\right)}{\underset{︸}{\sum_{d=1}^{t-t_{0}}R_{0}\left(d\right)N\left(t-d\right)}},\forall t\geq t_{0},$$

where (I) is the number of infected produced directly by the $I_{0}$ infected individuals already present on day $t_{0}$, and (II) is the number of new infected individuals at time *t* produced by the new infected individuals since day $t_{0}$.

If we consider the first terms of the discrete time Volterra Equation (6), we obtain

$$\begin{array}{cc} N(t_{0}) & =R_{0}\left(0\right)I_{0},\;\\ N(t_{0}+1) & =R_{0}\left(1\right)I_{0}+R_{0}\left(1\right)N\left(t_{0}\right),\;\\ N(t_{0}+2) & =R_{0}\left(2\right)I_{0}+R_{0}\left(2\right)N\left(t_{0}\right)+R_{0}\left(1\right)N\left(t_{0}+1\right),\;\\ N(t_{0}+3) & =R_{0}\left(3\right)I_{0}+R_{0}\left(3\right)N\left(t_{0}\right)+R_{0}\left(2\right)N\left(t_{0}+1\right)+R_{0}\left(1\right)N\left(t_{0}+2\right),\;\\ \;\vdots \end{array}$$

In practice, we can assume that $R_{0}\left(0\right)=0$ since infected individuals are not infectious immediately after being infected. Under this additional assumption, we obtain the system

$$\begin{array}{cc} N(t_{0}) & =0,\;\\ N(t_{0}+1) & =R_{0}\left(1\right)I_{0},\;\\ N(t_{0}+2) & =R_{0}\left(2\right)I_{0}+R_{0}\left(1\right)N\left(t_{0}+1\right),\;\\ N(t_{0}+3) & =R_{0}\left(3\right)I_{0}+R_{0}\left(2\right)N\left(t_{0}+1\right)+R_{0}\left(1\right)N\left(t_{0}+2\right),\;\\ \;\vdots \end{array}$$

Therefore, (6) can be rewritten as a scalar delay difference equation

(7) $$N\left(t\right)=R_{0}\left(1\right)N\left(t-1\right)+\ldots +R_{0}\left(t-t_{0}-1\right)N\left(t_{0}+1\right)+R_{0}\left(t-t_{0}\right)I_{0},\forall t\geq t_{0}.$$

Assume that the infectious period is *n* days. That is

$$R_{0}\left(a\right)=0,\forall a\geq n+1.$$

Then by defining $t_{1}=t_{0}+n+1$, Equation (6) becomes

(8) $$N\left(t\right)=\sum_{d=1}^{n}R_{0}\left(d\right)N\left(t-d\right),\forall t\geq t_{1},$$

with the initial values

(9) $$N\left(t\right)=N_{0}\left(t\right),\forall t\in \left[t_{1}-n,t_{1}\right].$$

The goal of this article is to understand how to identify the daily reproduction numbers $d\in \left\{1,\cdots ,n\right\}\mapsto R_{0}\left(d\right)$ in (8) knowing $t\in \left[t_{1},t_{2}\right]\mapsto N\left(t\right)$ on some finite time interval. This problem is particularly important to derive the average dynamic of infection at the level of a single patient.

One of the aims of this paper is to investigate the variations of the daily reproduction number $d\in \left\{1,\cdots ,n\right\}\mapsto R_{0}\left(d\right)$ during the period of contagiousness of infectious individuals.

This is not the case in influenza, as shown in simulated data [3] and in real infected animals, where we observe a U-shaped evolution of their viral load and symptoms as their body temperature during their contagiousness period. From there, it is possible to suspect a U-shaped variation in their ability to emit (aerosol transmission) the virus and, therefore, to contaminate it [4].

After the first asymptomatic period (without contagiousness), the daily reproduction number increases. After one to three days, this number decreases due to the action of the first defense of the innate immune system. But, the virus passes over this first immune defense, and the daily reproduction number increases again before the action of the second adaptive immune system. Then, after two to four days, the second adaptive immune response becomes fully effective. The combination of these biological mechanisms causes the daily reproduction numbers’ U- or M-shaped curve.

The literature about parameters identification for epidemic models with age of infection can be divided into two groups of articles depending on the assumptions made. The first group assumes that d↦Γ(d) is a given function and estimates the time dependent transmission rate t↦τ(t). As a consequence, they obtain the instantaneous (daily or effective) reproduction number, which is

$$R_{0}\left(t\right)=\tau \left(t\right)S\left(t\right)\sum_{d=1}^{n}\Gamma \left(d\right).$$

We refer to [5,6,7,8,9,10,11,12] (and references therein) for more results about this subject.

The second group corresponds to the assumptions considered here. That is, we assume that $t\mapsto \tau \left(t\right)=\tau_{0}$ and $t\mapsto S\left(t\right)=S_{0}$ are constant functions (over a short period of time) and estimate the daily reproduction number. That is the case for the discrete time model in [13] and more recently for the continuous time model in [1]. The major default in [13] is that the estimated $d\mapsto R_{0}\left(d\right)$ does not remain positive. We will have the same problem is Section 3.1 when we will use the full spectrum. In Section 3.2, to solve this problem, we introduce a method using the dominant and secondary eigenvalue only.

**Figure 1:**
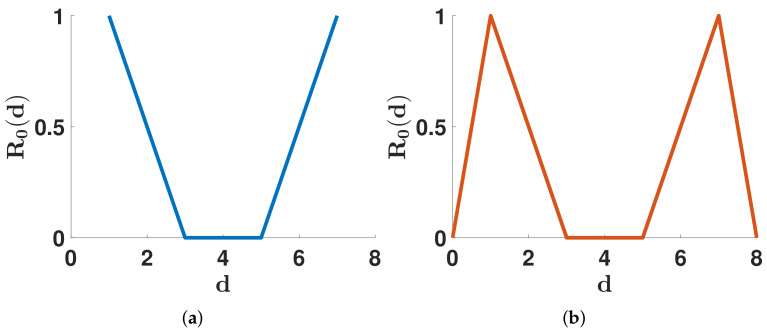
In this figure, we illustrate the notion of U shape distribution in (**a**) and M shape distribution in (**b**). Recall that $R_{0}\left(d\right)$ represents the ability of patients to transmit the pathogen after *d* days since they were infected. The U shape or M shape distribution means that patients can transmit the pathogen since the beginning of their infection. Then they become less infectious in the middle of the infected period. Finally, they become infectious again at the end of the infected period. The only difference between U and M shape distribution is to include days 0 and 8 and $R_{0}\left(0\right)=R_{0}\left(8\right)=0$ in the plot.

This article aims to investigate the shape of the distribution $d\mapsto R_{0}\left(d\right)$ from the data of COVID-19. In Figure 1, we illustrate the notion of U- or M-shaped distribution.

**Figure 2:**
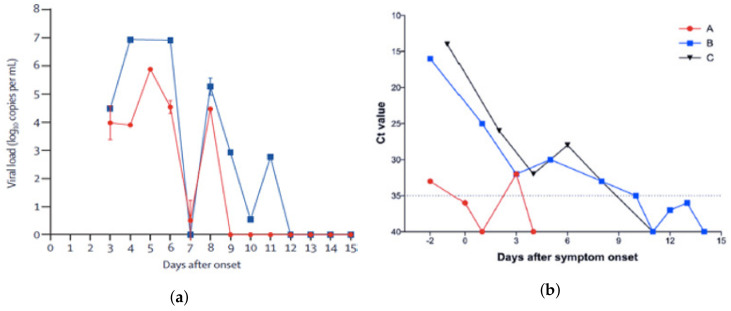
Viral load in COVID-19 real patients [14]. In (**a**), the red curve corresponds to the throat swab and the blue curve corresponds to the sputum. In (**b**), the curves correspond to several patients (A), (B), and (C).

The U and M shape distribution are well known in the context of influenza [3,4]. In Figure 2, we present some figures reflecting patients’ viral load for COVID-19.

Such U shape has not yet been systematically studied in COVID-19 data, but observations of the evolution of the viral load have been done in some patients and show this U shape. Figure 2 shows such a U-shaped evolution for the viral load in real patients [14].

The present work is directly connected to the original work of Peter Whittle in 1951 [15,16], who introduced the Auto Regressive Moving Average (ARMA) model, after the seminal paper on time series by N. Wiener [17],

(10) $$N\left(t\right)=\underset{\;\mathrm{Auto}\;\mathrm{regressive}\;\mathrm{part}}{\underset{︸}{K(1)N(t-1)+K(2)N(t-2)+\ldots +K(n)N(t-n)}}+\underset{\;\mathrm{Moving}\;\mathrm{average}\;\mathrm{part}}{\underset{︸}{w(t)}},$$

where N(t) is the size at time *t* of the population whose growth is forecasted, the kernel d↦K(d) has real values, *n* is the regression order, and here w(t) stands for a noise. Equation (10) has been extensively studied under the denomination of ARMA models by many authors [18,19,20,21,22,23,24].

Here, we propose a new approach based on the spectral properties of the population growth equation to capture information from data. Our goal is to estimate the shape of the daily reproduction numbers $d\mapsto R_{0}\left(d\right)$. Spectral methods are not new (see Priestley [20,25]), but it usually refers to Fourier transform with frequencies associated to various periods, corresponding to a fundamental period and its sub-multiples (harmonics). If we consider the auto regressive part only, the spectrum of the delay difference equation is determined by its characteristic equation

$$\lambda^{n}=K\left(1\right)\lambda^{n-1}+K\left(2\right)\lambda^{n-2}+\ldots +K\left(n-1\right)\lambda +K\left(n\right).$$

The main idea in this article is to use these eigenvalues $\lambda_{1},\lambda_{2},\ldots ,\lambda_{n}\in C$ (i.e., the solution of the characteristic equation) to identify the parameters K(1),K(2),…,K(n). The eigenvalues $\lambda_{1},\lambda_{2},\ldots ,\lambda_{n}\in C$ are estimated by some separated method. In Section 2, we will see that when all the eigenvalues are non null and separated two by two, then we can compute the parameters K(1),K(2),…,K(n) by using the eigenvalues only.

The idea of using eigenvalues in population dynamics goes back to Malthus [26], who, in 1798, first identified in a mixture of populations the one that would impose itself on the others, determined through the exponential growth of the largest exponent—this leading exponent having been called Malthusian parameter by Fisher [27]. The Malthusian growth seeming unrealistic, the saturation logistic term was introduced further by Lambert [28], and then extending the initial work by Euler [29], Lotka [30], Leslie [31], and Hahn [32] gave the current matrix form of the discrete population growth equations.

However, as far as we know, estimating the subdominant eigenvalues to characterize the system is new. So the key idea of this work is to use the dominant eigenvalue $\lambda_{1}$ and also the following pair of complex conjugated eigenvalues $\lambda_{2},{\bar{\lambda }}_{2}$ as an estimator to reconstruct the kernel of the auto regressive part.

This work is motivated by the times series provided by data of the daily numbers of reported cases of COVID-19. During the COVID-19 pandemic, most people viewed the oscillations around the exponential growth at the beginning of an epidemic wave as the default in reporting the data. The residual is probably partly due to the reporting data process (random noise). Nevertheless, a significant remaining part of such oscillations could be connected to the infection dynamic at the level of a single average patient. Eventually, the central question we try to address here is: Is there some hidden information in the signal around the exponential tendency for COVID-19 data? So we consider the early stage of an epidemic phase, and we try to exploit the oscillations around the tendency in order to reconstruct the infection dynamic at the level of a single average patient.

We start by investigating the connection between a signal decomposed into a sum of damped or amplified oscillations and a renewal equation. The prototype example we have in mind is the following:

$$N\left(t\right)=A_{1}e^{\alpha_{1}t}+e^{\alpha_{2}t}\left[A_{2}\cos\left(\omega_{2}t\right)+B_{2}\sin\left(\omega_{2}t\right)\right]+C,\;\forall t\geq t_{1}-n,$$

where $A_{1},A_{2},A_{3}\in R$, $\alpha_{1}>0$, $\alpha_{2}\in R$, and $\omega_{2}>0$.

**Figure 3:**
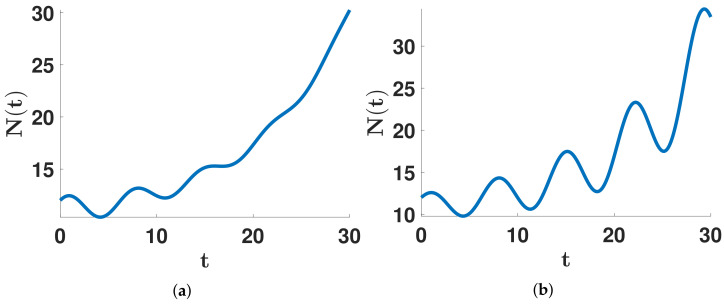
We plot an exponentially growing function with (**a**) damped oscillations and (**b**) amplified oscillations.

In Figure 3, we illustrate a growing function with damped oscillations (i.e., $\alpha_{2}<0$) and amplified oscillations (i.e., $\alpha_{2}>0$). It is clear from Figure 3 that a periodic function can not represent such a signal, and extending such a signal by periodicity would be artificial. Indeed, the Fourier decomposition would only provide purely imaginary eigenvalues that would exclude a continuation of the exponential growth (i.e., eigenvalues with non-zero real parts). To apply wavelets theory (see, for example, in [33]), we need to extend the data for negative times by symmetry with respect to the initial time t=0, and we need a decreasing function ($\alpha_{1}<0$ and $\alpha_{2}<0$).

Here, we are more interested in the model resulting from the data (i.e., $R_{0}\left(d\right)\geq 0$, ∀d=1,…,n) than in the fit to the data. The major problem with the Fourier method is that this method provides only eigenvalues with zero real parts (that is due to the periodicity required for this method). Such eigenvalues are well adapted to a periodic signal, but this is not suitable to describe, for example, an ever-growing function (as in Figure 3). Consequently, the Fourier method is not well adapted to derive non-negative daily reproduction numbers (i.e., $R_{0}\left(d\right)\geq 0,\forall d=1,\ldots ,n$).

Previous analogous approaches can be found in the seismic data modeling and statistical literature, like the Wiener–Levinson predictive deconvolution (Robinson [34], Peacock and Treitel [35], Robinson and Treitel [36]), which intends to estimate the minimum phase wavelet in the data, in particular in the case where the relatively weak sampling does not make it possible to affirm the Gaussian character of the errors (Walden and Hosken [37]). If the Gaussian character of the errors can be proven, another similar approach is that of the Geometric Brownian Motion (GBM) processes (Vinod et al. [38]) used, for example, in the analysis of financial data (Ritschel et al. [39]), which are based on the model of the solution of a stochastic differential equation, multiplied by a periodic component with a Gaussian noise.

The structure of this paper is as follows: Section 2 is devoted to the materials and methods. We recall some notions of matrices and spectra. We also present some phenomenological models that will be compared to the data. Section 3 contains the results. We fit the phenomenological models to the cumulative numbers of reported cases in Japan over 10 days and 30 days. We use the eigenvalues derived from the phenomenological model, and we identify the daily reproduction numbers by using: (1) all the spectrum (see Appendix B) and (2) part of the spectrum. The last section of the paper is devoted to the discussion and the conclusion. We present in the Appendices all the mathematical aspects of the paper (see Appendix A, Appendix B, Appendix C and Appendix D).

## 2. Materials and Methods

### 2.1. Identification of the Model

The Leslie matrix associated to the difference Equation (8) is
(11)
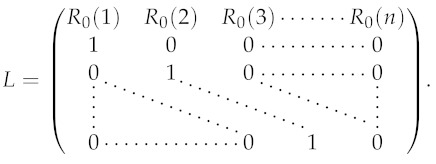


The **characteristic equation** of (11) is

(12) $$\lambda^{n}=\sum_{d=1}^{n}R_{0}\left(d\right)\lambda^{n-d},$$

for λ∈C, which is equivalent to (whenever λ≠0)

(13) $$1=\sum_{d=1}^{n}R_{0}\left(d\right)\lambda^{-d}.$$

The complex numbers satisfying the characteristic equation are called the eigenvalues of *L*.

In Appendix A and Appendix B, we discuss the identification problem of the daily reproduction numbers $R_{0}\left(1\right),\ldots ,R_{0}\left(n\right)$ by using the eigenvalues of *L*. The main identification result of Appendix B corresponds to the formula (A3).

**Definition** **1.** *We will say that L is a **Markovian Leslie matrix** if all the values* $d\in \left[1,n\right]\mapsto R_{0}\left(d\right)$ *are non negative, and*

$$\sum_{d=1}^{n}R_{0}\left(d\right)=1.$$

### 2.2. Phenomenological Model to Fit the Cumulative and the Daily Numbers of Reported Case Data

Due to Lemma A1 below, we propose the following phenomenological model to represent the data

(14) $$\mathrm{CR}\left(t\right)={\mathrm{CR}}_{1}e^{\lambda_{1}t}+{\mathrm{CR}}_{2}e^{\lambda_{2}t}+{\mathrm{CR}}_{3}e^{\lambda_{3}t}+\ldots +{\mathrm{CR}}_{m}e^{\lambda_{m}t},$$

where ${\mathrm{CR}}_{1},\ldots ,{\mathrm{CR}}_{m}\in C$ are non null, $\lambda_{1}=\alpha_{1}+i\omega_{1},\ldots ,\lambda_{m}=\alpha_{m}+i\omega_{m}\in C$ are pairwise distinct, and m≤n.

**Remark** **1.** *In the above formula, we allow the constant terms whenever*$\lambda_{n}=0$.

Assuming that the unit of time is one day, we have the following relationship between the cumulative number of cases CR(t) and the daily number of cases N(t)

$$\mathrm{CR}\left(t\right)=\mathrm{CR}\left(t_{0}\right)+\int_{t_{0}}^{t}N\left(\sigma \right)d\sigma .$$

We deduce that the daily number of reported cases has the following form

$$N\left(t\right)=N_{1}e^{\lambda_{1}t}+N_{2}e^{\lambda_{2}t}+N_{3}e^{\lambda_{3}t}+\ldots +N_{m}e^{\lambda_{m}t},$$

where $N_{1},\ldots ,N_{m}\in C$ are non null, and $\lambda_{1},\ldots ,\lambda_{m}\in C$ are the same as in (14), and m≤n.

Since N(t) is obtained from CR(t) by computing the first derivative, we have the following relationship

$$N_{k}={\mathrm{CR}}_{k}\lambda_{k},\forall k=1,\ldots ,m.$$

**Remark** **2.** *For the daily number of cases data*t↦N(t)*only a few eigenvalues will be tractable. For example, in Section 3.3, we will consider the following extension*

$$N\left(t\right)=N_{1}e^{\lambda_{1}t}+N_{2}e^{\lambda_{2}t}+N_{3}e^{\lambda_{3}t}+N_{3}e^{\lambda_{3}t}+N_{4}e^{\lambda_{4}t}+w\left(t\right)$$

*where*w(t)*will contain*$N_{5}e^{\lambda_{5}t}+\ldots +N_{m}e^{\lambda_{m}t}$*merged together with some random term*.

**Remark** **3.** *The identification of the eigenvalues*$\lambda_{1},\ldots ,\lambda_{m}$*as parameters of the phenomenological model is discussed in Section 3.3. So far, this problem for a finite time interval seems to be open*.

We will first approach the data with the following phenomenological model.


Phenomenological model for the cumulative numbers of reported cases with λ>0
We start with a first eigenvalue $\lambda =e^{\alpha }>0$, for some α∈R. The phenomenological model used to fit the cumulative numbers of reported cases has the following form
(15)$$\mathrm{CR}\left(t\right)=Ae^{\alpha (t-t_{0})}+C,\;\mathrm{for}\;t\in \left[t_{0},+\infty \right),$$
where A∈R, α∈R, and C∈R are real numbers. For discrete times, it is equivalent to say that
(16)$\mathrm{CR}\left(n\right)=A\lambda^{n}+C,\;\mathrm{for}\;n=0,1,2,\ldots .$ By computing the first derivative of t↦CR(t), we obtain a model for the daily number of cases of the following form
(17)$$N\left(t\right)=A\;\alpha \;e^{\alpha (t-t_{0})},\;\mathrm{for}\;t\in \left[t_{0},+\infty \right).$$

Once the best fit of the above phenomenological model to the data is obtained, we can subtract this model to the data $t\mapsto {\mathrm{CR}}_{\mathrm{Data}}\left(t\right)$, then we obtain a first residual

$$\mathrm{Residual}\left(t\right)={\mathrm{CR}}_{\mathrm{Data}}\left(t\right)-\mathrm{CR}\left(t\right).$$

Next we will approach the residual with the following phenomenological model.



Phenomenological model for the cumulative numbers of reported cases with λ∈C
Assume that the eigenvalues are two conjugated complex numbers $\lambda =e^{\alpha \pm i\omega }\in C$, for some α∈R and ω≥0. The phenomenological model used to fit the cumulative numbers of reported cases has the following form
(18)$$\mathrm{CR}\left(t\right)=e^{\alpha (t-t_{0})}\left[A\cos\left(\omega \left(t-t_{0}\right)\right)+B\sin\left(\omega \left(t-t_{0}\right)\right)\right]+C,\;\mathrm{for}\;t\in \left[t_{0},+\infty \right),$$
where α∈R, A∈R, B∈R, C∈R, and ω≥0 are four real numbers. For discrete times, it is equivalent to say that
(19)$\mathrm{CR}\left(n\right)=\frac{A-iB}{2}\lambda^{n}+\frac{A+iB}{2}{\bar{\lambda }}^{n}+C,\;\mathrm{for}\;n=0,1,2,\ldots .$ By computing the first derivative of t↦CR(t), we obtain a model for the daily number of cases of the following form
(20)$$\begin{array}{c} N\left(t\right)=e^{\alpha (t-t_{0})}\left[\hat{A}\cos\left(\omega \left(t-t_{0}\right)\right)+\hat{B}\sin\left(\omega \left(t-t_{0}\right)\right)\right],\;\mathrm{for}\;t\in \left[t_{0},+\infty \right), \end{array}$$
where
(21)$$\left\{\begin{array}{c} \hat{A}=\alpha A+\omega B\\ \hat{B}=-\omega A+\alpha B \end{array}\right.\Leftrightarrow \left\{\begin{array}{c} A=\frac{\alpha \hat{A}-\omega \hat{B}}{\omega^{2}+\alpha^{2}}\\ B=\frac{\omega \hat{A}+\alpha \hat{B}}{\omega^{2}+\alpha^{2}}\; \end{array}\right..$$

**Remark** **4.** *When*ω=0*in *(18)*, we obtain the previous model *(15)*.*

### 2.3. Cumulative and Daily Number of Reported Cases for COVID-19 in Japan

**Figure 4:**
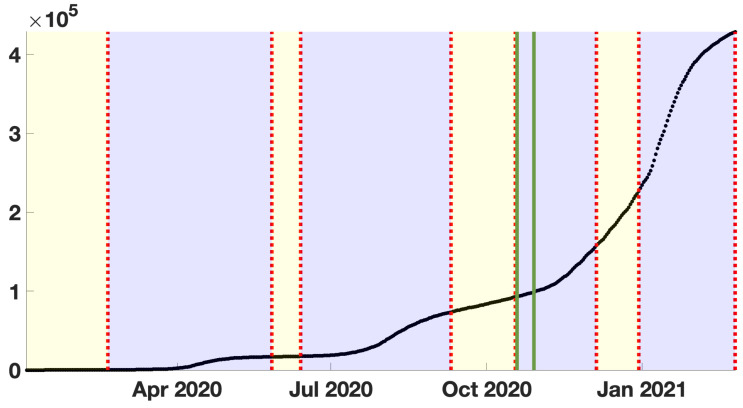

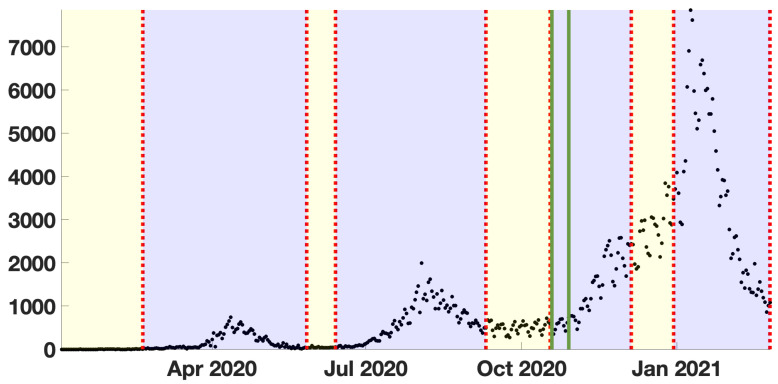
In this figure, we plot the daily number of reported cases for COVID-19 in Japan.

Here we use cumulative numbers of reported cases for COVID-19 in Japan taken from the WHO [40]. The data show a succession of epidemic waves (blue background color regions) followed by endemic periods (yellow background color regions). In Figure 4, black dots represent the data. The blue background color regions correspond to epidemic phases, and the yellow background color region to endemic phases. The region of interest to apply the method is between 19 October and 29 October 2020. This region is marked with light green vertical lines in the figure.

## 3. Results

### 3.1. Methods Applied to Ten Days Data

In this section, we will fit the phenomenological model (15) or (18) to the cumulative numbers of reported cases presented in the previous subsection. We consider a period of 10 days since the beginning of the third epidemic wave of COVID-19 in Japan. The period goes from 19 to 29 October 2020.

**Figure 5:**
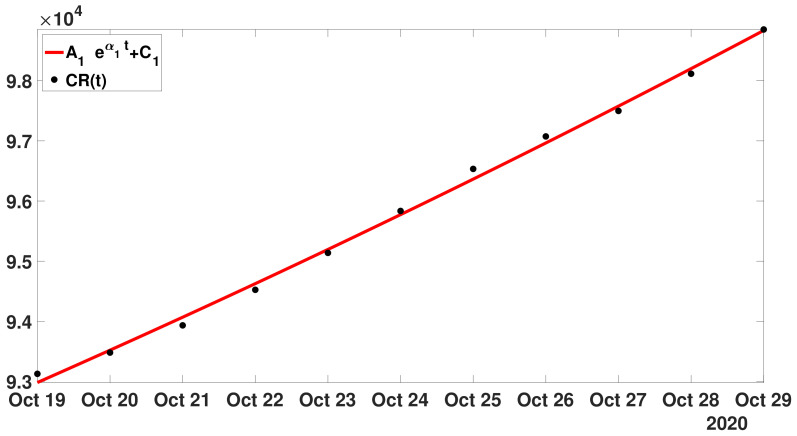
In this figure, the black dots correspond to the cumulative numbers of reported cases of COVID-19 in Japan between 19 October and 29 October 2020 (black dots). The red curve corresponds to the best fit of model (15) to the cumulative numbers of reported cases.

**Step** **1:** In Figure 5, we fit an exponential function (15) to the cumulative number of reported cases of COVID-19 in Japan between 19 and 29 October 2020.

In Figure 5, the best fit of model (15) is obtained for

$$A_{1}=2.8810^{4},C_{1}=6.4210^{4},\;\mathrm{and}\;\alpha_{1}=0.02.$$

Hence,

$$\lambda_{1}=\exp\left(\alpha_{1}\right)=1.02.$$

**Step** **2:** Next, we consider the residual left after the previous fit,
  $${\mathrm{Residual}}_{1}\left(t\right)=\mathrm{CR}\left(t\right)-\left[A_{1}e^{\alpha_{1}t}+C_{1}\right].$$

**Figure 6:**
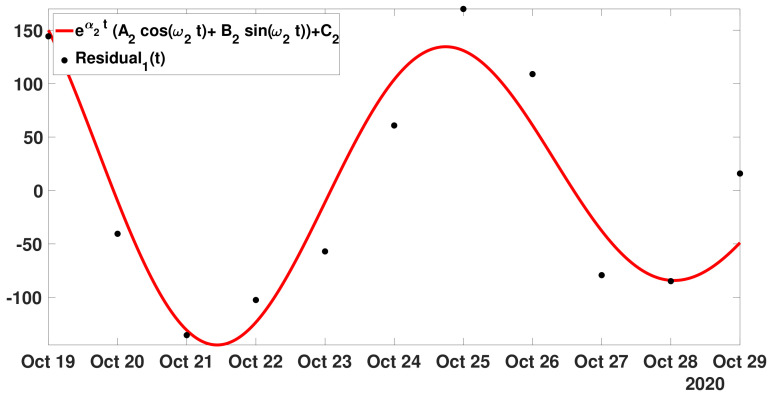
In this figure, the black dots correspond to the function $t\mapsto {\mathrm{Residual}}_{1}\left(t\right)$ from 19 October and 29 October 2020 (black dots). The red curve corresponds to the best fit of model (18) to ${\mathrm{Residual}}_{1}\left(t\right)$.

In Figure 6, we fit the model (18) to the first residual function $t\mapsto {\mathrm{Residual}}_{1}\left(t\right)$.

In Figure 6, the best fit of model (18) (i.e., minimizing the sum-of-squares error) is obtained for

$$A_{2}=138.16,B_{2}=-127.36,C_{2}=11.88,\alpha_{2}=-0.07,\;\mathrm{and}\;\omega_{2}=0.95.$$

The period associated to $\omega_{2}$ is equal to $P_{2}=\frac{2\pi }{\omega_{2}}=6.609$ days. This periodic phenomenon was observed in many countries (see for example [41]). Here,

$$\lambda_{2}=\exp\left(\alpha_{2}+i\;\omega_{2}\right)=0.54+0.76\;i,$$

$$\lambda_{3}=\exp\left(\alpha_{2}-i\;\omega_{2}\right)=0.54-0.76\;i.$$

By using

$$M=(\begin{array}{ccc} \lambda_{1}^{-1} & \lambda_{1}^{-2} & \lambda_{1}^{-3}\\ \lambda_{2}^{-1} & \lambda_{2}^{-2} & \lambda_{2}^{-3}\\ \lambda_{3}^{-1} & \lambda_{3}^{-2} & \lambda_{3}^{-3} \end{array}),$$

in (A3) below, with n=3, we obtain

(22) $$(\begin{array}{c} R_{0}\left(1\right)\\ R_{0}\left(2\right)\\ R_{0}\left(3\right) \end{array})=(\begin{array}{c} 2.09\\ -1.96\\ 0.87 \end{array}).$$

Since

det(M)=1.78i,

therefore, the components of $M^{-1}$ are not too large, and the above result should not be too sensitive to the stochastic errors. The main problem in (22) is the second component −1.9625, which is not making sense in this context.

### 3.2. Spectral Truncation Method Applied to Ten Days Data

In the previous subsection, the first two fits make perfect sense. However, adding more fits would be questionable because they become more and more random after a few steps. We could alternatively continue to fit the rest by using our phenomenological model, which would provide new eigenvalues.

The major problem in the previous section is that when we apply formula (A3) with all the eigenvalues, we obtain some $R_{0}\left(1\right),\ldots ,R_{0}\left(n\right)$ with negative values. Instead here, we increase the dimension *n* of *L*, and we use only the eigenvalues $\lambda_{1},\lambda_{2},\lambda_{3}$.

#### 3.2.1. Re-Normalizing Procedure

Assume that $\lambda_{1}\neq 1$ then by

$$\bar{N}\left(t\right)=\frac{N(t)}{\lambda_{1}^{t}}\Leftrightarrow N\left(t\right)=\lambda_{1}^{t}\bar{N}\left(t\right)$$

where t↦N(t) is a solution of (8), we obtain the following normalized equation

$$\lambda_{1}^{t}\bar{N}\left(t\right)=\sum_{d=1}^{n}R_{0}\left(d\right)\lambda_{1}^{t-d}\bar{N}\left(t-d\right),\forall t\geq t_{1},$$

and by dividing the above equation by $\lambda_{1}^{t}$ we obtain

$$\bar{N}\left(t\right)=\sum_{d=1}^{n}{\bar{R}}_{0}\left(d\right)\;\bar{N}\left(t-d\right),\forall t\geq t_{1}.$$

where

(23) $$R_{0}\left(d\right)={\bar{R}}_{0}\left(d\right)\lambda_{1}^{d},\forall d=1,\ldots ,n.$$

By using the procedure, we can always fix the dominant eigenvalue of *L* to 1 by imposing that *L* is Markovian (see Definition 1). Then we use the following re-normalizing procedure for the eigenvalues

$$\lambda_{1}^{🟉}=\lambda_{1}/\lambda_{1}=1,\lambda_{2}^{🟉}=\lambda_{2}/\lambda_{1}=0.53+0.74\;i,\;\mathrm{and}\;\lambda_{3}^{🟉}=0.53-0.74\;i.$$

**Figure 7:**
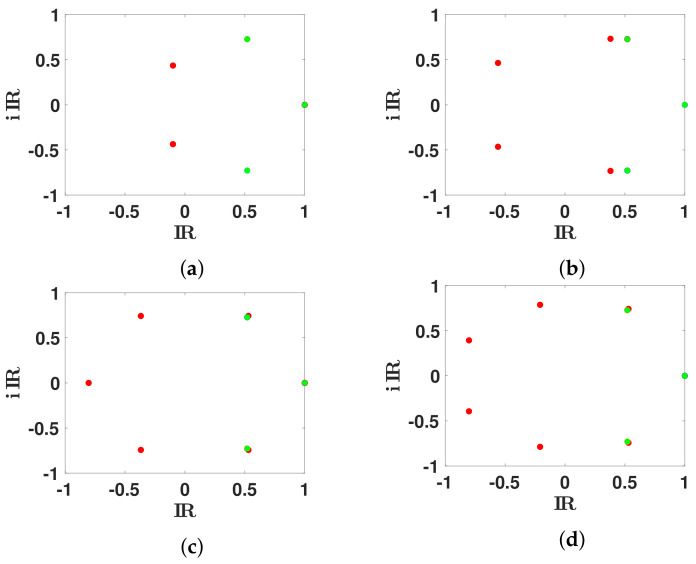
We plot the spectrum of the Markovian Leslie matrices *L* (red dots) when n=3,5,6,7, (respectively in (**a**–**d**)) giving the best match to the secondary eigenvalues $\lambda_{2}^{🟉}$ and $\lambda_{3}^{🟉}$ (green dots). We observe that the best fit of the two secondary eigenvalues remain far away from $\lambda_{2}^{🟉}$ and $\lambda_{3}^{🟉}$ for n=3, then get closer for n=5, and are very close for n=6 and n=7.

In Figure 7, we fit these eigenvalues $\lambda_{2}^{🟉}$ and $\lambda_{3}^{🟉}$ with the spectrum of Markovian Leslie matrices *L* on a mesh. We observe that the fit improves when the dimension of *L* increases.

**Figure 8:**
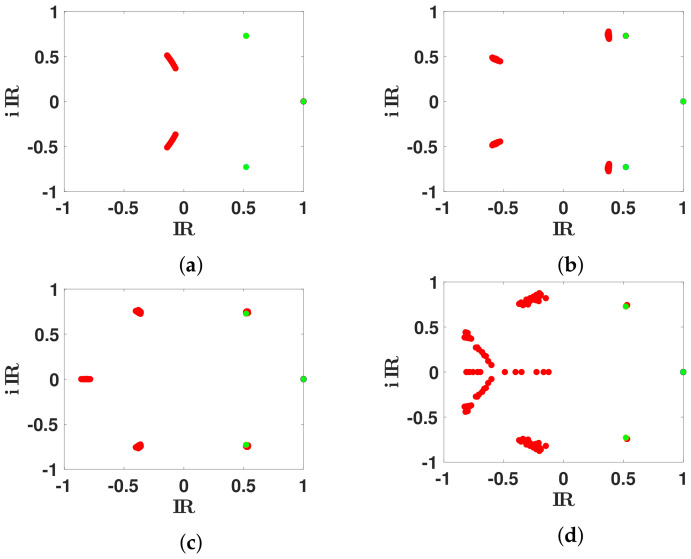
We plot the spectrum of the Leslie matrix *L* (red dots) when n=3,5,6,7, (respectively in (**a**–**d**)) giving the best match to the secondary eigenvalues $\lambda_{2}^{🟉}$ and $\lambda_{3}^{🟉}$ (green dots). The red dots correspond to the spectrum of *L* for all the possible matrices *L*, having their second pair of eigenvalues close to the minimal distance to $\lambda_{2}^{🟉}$ and $\lambda_{3}^{🟉}$.

In Figure 8, we observe that, for $n\in \left\{3,5,6\right\}$, there is a unique set of eigenvalues $\lambda_{1},\lambda_{2},\lambda_{3},\ldots ,\lambda_{n}$ of *L* (classified with decreasing real part) minimizing the distance $|\lambda_{2}^{🟉}-\lambda_{2}|$ and $|\lambda_{3}^{🟉}-\lambda_{3}|$. This is no longer true for n=7.

#### 3.2.2. Daily Basic Reproduction Numbers

**Figure 9:**
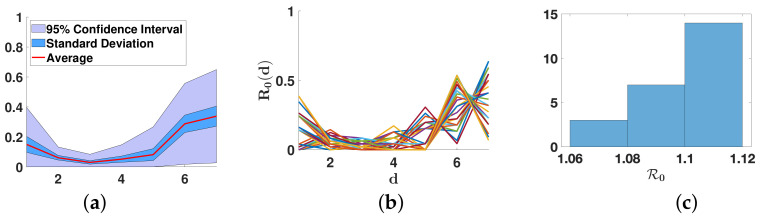
In this figure, we use the distributions $d\mapsto R_{0}\left(d\right)$ minimizing the distance $|\lambda_{2}^{🟉}-\lambda_{2}|$ and $|\lambda_{3}^{🟉}-\lambda_{3}|$ whenever n=7. In (**a**), we plot the average distribution $d\mapsto R_{0}\left(d\right)$ (red curve), standard deviation (blue region), and 95% confidence interval (light blue region). In (**b**), we plot the 24 distributions $d\mapsto R_{0}\left(d\right)$. In (**c**), we give a histogram with the multiple values of $R_{0}=\sum_{d=1}^{n}R_{0}\left(d\right)$. We observe that some of the $d\mapsto R_{0}\left(d\right)$ are similar to the case n=6, with a maximum on day d=6, but on average the maximum value is on day 7.

In Figure 9, we plot the average distribution $d\mapsto R_{0}\left(d\right)$, standard deviation (blue region), and 95% confidence interval.

**Figure 10:**
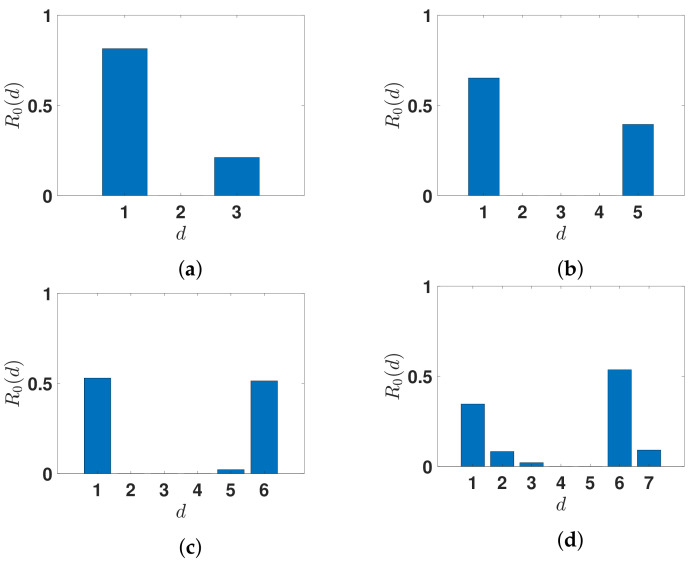
We plot the daily basic reproduction numbers $R_{0}\left(d\right)$ obtained for n=3 in (**a**), n=5 in (**b**), n=6 in (**c**), and n=7 in (**d**). The distribution for n=7 corresponds to the red curve in Figure 9.

In Figure 10, we plot the daily basic reproduction numbers $R_{0}\left(d\right)$.

We can notice that following [42], the effective $R_{0}$ is between 1.06 and 1.14 on 19 October 2020, in Japan.

#### 3.2.3. Applying the Model to Daily Number of Reported Cases

The model used to run the simulations is the following

(24) $$N\left(t\right)=\sum_{d=1}^{6}R_{0}\left(d\right)N\left(t-d\right),\forall t\geq t_{0}+6,$$

and according to the formula (17) and (20), with the initial condition

(25) $$N\left(t\right)=A_{1}\ln\left(\lambda_{1}\right)\lambda_{1}^{t}+e^{\alpha_{2}t}\left[{\hat{A}}_{2}\cos\left(\omega_{2}t\right)+{\hat{B}}_{2}\sin\left(\omega_{2}t\right)\right],\forall t=t_{0},t_{0}+1,\ldots ,t_{0}+5,$$

with

(26) $${\hat{A}}_{2}=\alpha_{2}A_{2}+\omega_{2}B_{2}\;\mathrm{and}\;{\hat{B}}_{2}=-\omega_{2}A_{2}+\alpha_{2}B_{2}.$$

In (24)–(26) we use the parameter values estimated in Section 3.1.

**Figure 11:**
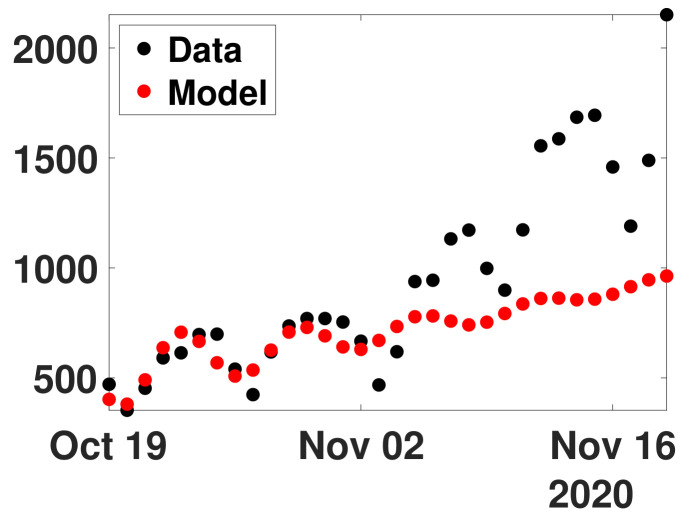
In this figure, we plot the daily number of reported cases data from October 19 and November 19, 2020 (black dots) and from model (24) and (25) with the values of $R_{0}\left(d\right)$ obtained in Figure 10c (red dots).

In Figure 11, we plot the daily number of reported cases data from October 19 to November 19, 2020 (black dots) and from model (24) and (25) with the values of $R_{0}\left(d\right)$ obtained in Figure 10c (red dots).

### 3.3. Extension of the Spectral Truncation Method over One Month

**Figure 12:**
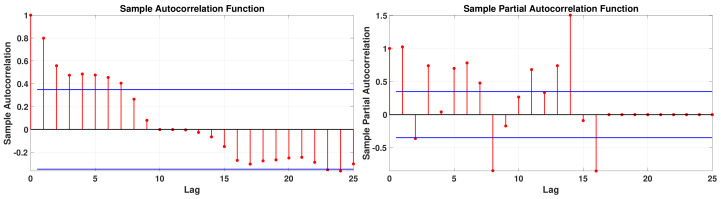
Autocorrelation Function (ACF) (left hand side) and Partial Autocorrelation Function (PACF) (right hand side) applied to the daily number of cases for Japan between 19 October and 19 November 2020.

In Figure 12, we apply respectively the AutoCorrelation Function (ACF) and Partial AutoCorrelation Function (PACF) to the daily number of cases for Japan from 19 October and 19 November 2020. It does not look like any standard cases. In the ACF, we observe the correlation is significant until 7 days, while in the PACF it is until 16 days.

**Figure 13:**
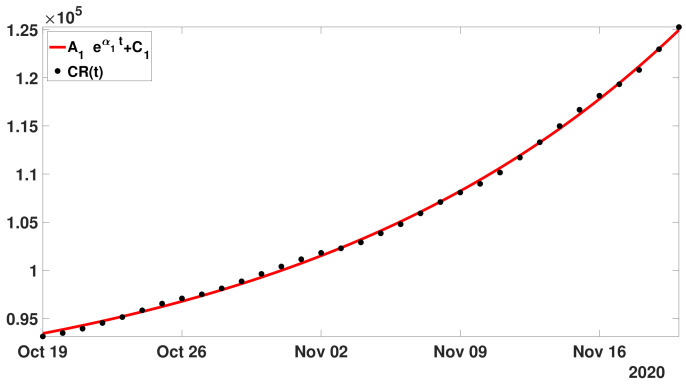
In this figure, we plot the cumulative number of reported cases data between 19 October and 19 November 2020 (black dots). We plot the best fit of the model (27) to the cumulative data (red curve).

**Step** **1:** In Figure 13, we fit the model
  (27) $$\phi_{1}\left(t\right)=A_{1}e^{\alpha_{1}\left(t-t_{0}\right)}+C_{1},$$
  with the cumulative number of reported cases data between 19 October and 19 November 2020.

We obtain the following parameter values for the best fit

(28) $$A_{1}=7.9310^{3},C_{1}=8.5510^{4},\;\mathrm{and}\;\alpha_{1}=0.05.$$

**Step** **2:** Next we define as before the first residual
  (29) $${\mathrm{Residual}}_{1}\left(t\right)=\mathrm{CR}\left(t\right)-A_{1}e^{\alpha_{1}\left(t-t_{0}\right)}+C_{1},$$
  and we fit the ${\mathrm{Residual}}_{1}\left(t\right)$ with the model
  (30) $$\begin{array}{cc} \phi_{2}\left(t\right) & =e^{\alpha_{2}\left(t-t_{0}\right)}\left[A_{2}\cos\left(\omega_{2}\left(t-t_{0}\right)\right)+B_{2}\sin\left(\omega_{2}\left(t-t_{0}\right)\right)\right]\;\\ \; & \;+e^{\alpha_{3}\left(t-t_{0}\right)}\left[A_{3}\cos\left(\omega_{3}\left(t-t_{0}\right)\right)+B_{3}\sin\left(\omega_{3}\left(t-t_{0}\right)\right)\right]+C_{2}. \end{array}$$

**Figure 14:**
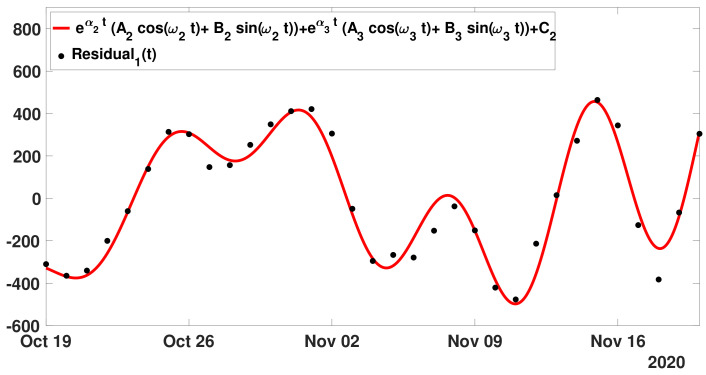
In this figure, we plot the cumulative number of reported cases data between 19 October and 19 November 2020 (black dots). We plot the best fit of the model (30) to the cumulative data (red curve).

In Figure 14, we plot the cumulative number of reported cases data between 19 October and 19 November 2020.

The parameters of the phenomenological model $\phi_{2}\left(t\right)$ obtained for the best fit are the following

(31) $$A_{2}=55.21,B_{2}=-84.48,A_{3}=-391.57,B_{3}=88.79,C_{2}=7.68,$$

and

(32) $$\alpha_{2}=0.0501,\omega_{2}=0.91,\alpha_{3}=-0.02,\omega_{3}=0.3.$$

The periods associated to $\omega_{2}$ and $\omega_{3}$ are, respectively,

$$P_{2}=\frac{2\pi }{\omega_{2}}=6.92\;\mathrm{days},\;\mathrm{and}\;P_{3}=\frac{2\pi }{\omega_{3}}=21.24\;\mathrm{days}.$$

These periods are close multiples of 7 days.

**Remark** **5.** *It is important to note that the period*$P_{3}$*of *21* days is difficult to explain mechanically, but this value is the smallest value giving the best fit to the data. We tried to impose some upper bounds smaller than *21* days. In such a case,*$P_{3}$*is always replaced by the upper bound. This is true for all constraints less that *21* days, and for each constraint larger than *22* days, we obtain*$P_{3}=21.24$*days.*

**Remark** **6.** *It is important to note that* $\alpha_{1}=\alpha_{2}$ *. That is because, during the fit, we impose that* $\alpha_{2}\leq \alpha_{1}$ *and* $\alpha_{3}\leq \alpha_{1}$ *. That is the condition coming from the Perron Frobenius theorem, in order to obtain*

$$|\lambda_{2}\left|\leq \right|\lambda_{1}\left|\;and\;\right|\lambda_{3}\left|\leq \right|\lambda_{1}|.$$

*This condition is coming from the fact that* $\lambda_{1}$ *must be the spectral radius of L and* $\lambda_{2},\lambda_{3}$ *belong to the circle centered at 0 and with the radius equal to the spectral radius of L (i.e., with a modulus less or equal to* $\lambda_{1}$ *).*

**Eigenvalues associated to the model $\phi_{1}\left(t\right)$ and $\phi_{2}\left(t\right)$:** The first eigenvalue is

$$\lambda_{1}=e^{\alpha_{1}}=1.05.$$

The second pair of complex conjugated eigenvalues is

$$\lambda_{2}=e^{\alpha_{2}}\left[\cos\left(\omega_{2}\right)+i\sin\left(\omega_{2}\right)\right]=0.65+0.83\;i,$$

and

$$\lambda_{3}=e^{\alpha_{2}}\left[\cos\left(\omega_{2}\right)-i\sin\left(\omega_{2}\right)\right]=0.65-0.83\;i,$$

and the modulus of $\lambda_{2}$ is

$$|\lambda_{2}\left|=\right|\lambda_{3}|=e^{\alpha_{2}}=e^{\alpha_{1}}=\lambda_{1}=1.05.$$

The fourth eigenvalue is

$$\lambda_{4}=e^{\alpha_{3}}\left[\cos\left(\omega_{3}\right)+i\sin\left(\omega_{3}\right)\right]=0.94+0.29\;i,$$

and the fifth eigenvalue is its conjugate

$$\lambda_{5}=e^{\alpha_{3}}\left[\cos\left(\omega_{3}\right)-i\sin\left(\omega_{3}\right)\right]=0.94-0.29\;i,$$

and the modulus of $\lambda_{4}$ is

$$|\lambda_{4}\left|=\right|\lambda_{5}|=e^{\alpha_{3}}=0.98<1.05.$$

**Using $\lambda_{2}$ and $\lambda_{4}$ as an estimator:** Next we consider all the matrices *L* in which the component $R_{0}\left(d\right)$ is replaced by ${\bar{R}}_{0}\left(d\right)$, and we assume that

$$\sum_{d=1}^{n}{\bar{R}}_{0}\left(d\right)=1.$$

The dominant eigenvalue of *L* is 1, and we look for matrices such that the second eigenvalue of *L* is close to

$$\lambda_{2}^{🟉}=\lambda_{2}/\lambda_{1},$$

and the fourth eigenvalue of *L* is close to

$$\lambda_{4}^{🟉}=\lambda_{4}/\lambda_{1}.$$

For realizing this approach, we minimize the

$$\chi \left(L\right)=\max\left(d\left(\lambda_{2}^{🟉},\sigma \left(L\right)\right),d\left(\lambda_{4}^{🟉},\sigma \left(L\right)\right)\right)$$

where

$$d\left(\lambda_{2}^{🟉},\sigma \left(L\right)\right)=\min_{\lambda \in \sigma (L)}|\lambda_{2}^{🟉}-\lambda |,\;\mathrm{and}\;d\left(\lambda_{4}^{🟉},\sigma \left(L\right)\right)=\min_{\lambda \in \sigma (L)}\left|\lambda_{4}^{🟉}-\lambda \right|,$$

where σ(L) is the set of all eigenvalues of *L*.

**Figure 15:**
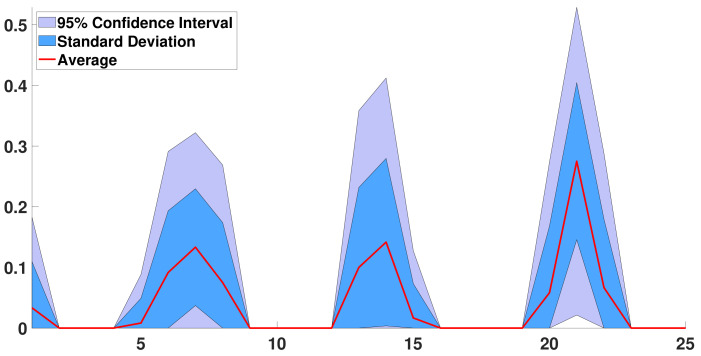
In this figure, we consider the case n=25. We plot the distributions of daily basic reproduction numbers $d\mapsto {\bar{R}}_{0}\left(d\right)$ corresponding to the distributions having some secondary eigenvalues and fourth eigenvalues at a distance less than $10^{-2}$ to the best match. The red curve is the average distribution $d\mapsto {\bar{R}}_{0}\left(d\right)$. The blue region corresponds to the standard deviation around the mean distribution. The light blue region corresponds to the 95% confidence interval.

In Figure 15, we consider the $d\mapsto {\bar{R}}_{0}\left(d\right)$ such that the corresponding maximum satisfies

$$\chi \left(L({\bar{R}}_{0})\right)\leq \underset{{\hat{R}}_{0}\geq 0:\sum {\hat{R}}_{0}\left(d\right)=1}{\inf}\chi \left(L({\hat{R}}_{0})\right)+10^{-2}.$$

We define

(33) $$R_{0}\left(d\right)={\bar{R}}_{0}\left(d\right)\lambda_{1}^{d},\forall d=1,\ldots ,n.$$

**Figure 16:**
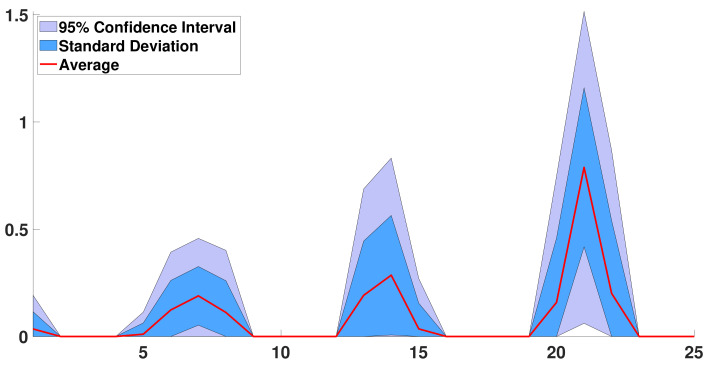
In this figure, we consider the case n=25. We plot the distributions of daily basic reproduction numbers $d\mapsto R_{0}\left(d\right)={\bar{R}}_{0}\left(d\right)\lambda_{1}^{d}$, where ${\bar{R}}_{0}\left(d\right)$ is the red curve in Figure 15.

In Figure 16, we obtain a good description of the dynamic of infection at the individual level that confirms the one obtained over shorter periods. As expected, the average patient first loses its ability to transmit the pathogen, and after decreasing by day 1 to day 4, $R_{0}\left(d\right)$ increases between day 4 and day 7. Day 7 is a maximum. After day 7, $R_{0}\left(d\right)$ decays until day 9. Then a second peak arises, with a maximum on the day 14. We could explain this second peak by supposing that an important transmission of pathogen still exists from day 12 to day 16. We also obtain a third from day 19 to 23 with a maximum value on day 21.

**Figure 17:**
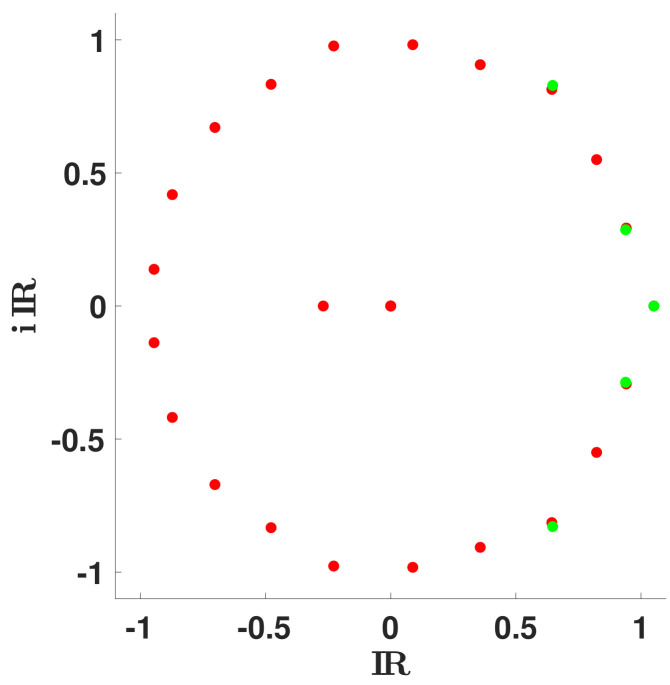
In this figure, we consider the case n=25. We plot the spectrum of the Leslie matrix *L* (red dots) when $d\mapsto {\bar{R}}_{0}\left(d\right)$ corresponds to the average distribution (i.e., the red curve in Figure 15).

In Figure 17, we plot the spectrum of the Leslie matrix *L* when $d\mapsto {\bar{R}}_{0}\left(d\right)$ corresponds to the average distribution (i.e., the red curve in Figure 15).

Recalling that, by definition, the basic reproduction number is

$$R_{0}=\sum_{d=1}^{n}R_{0}\left(d\right),$$

we obtain the sum of the daily reproduction numbers (red curve in Figure 16)

$$R_{0}=2.13.$$

**Figure 18:**
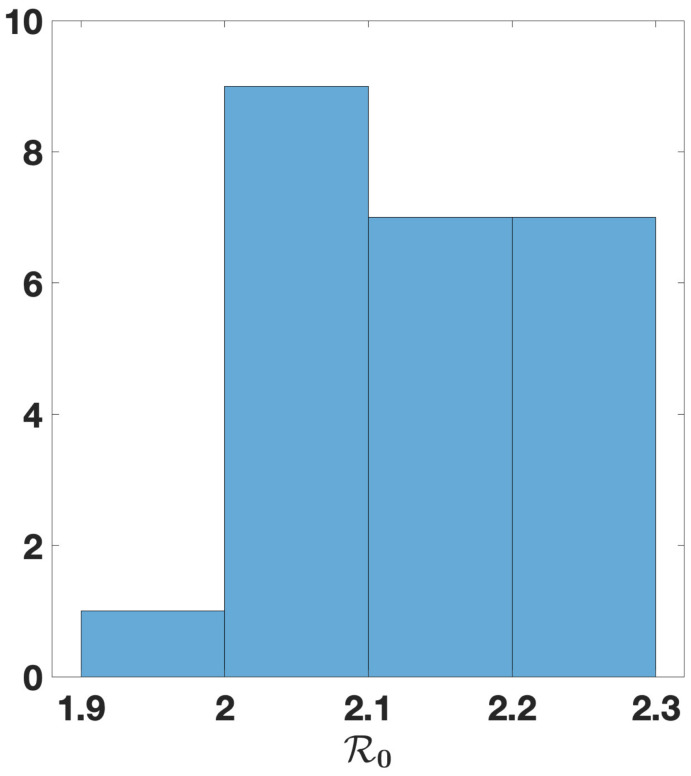
In this figure, we consider the case n=25, and we plot a histogram for the values of the basic reproduction number obtained by summing the distributions $d\mapsto R_{0}\left(d\right)$ from Figure 16.

In Figure 18, we plot a histogram for the values of the basic reproduction number obtained by summing the distributions $d\mapsto R_{0}\left(d\right)$ from Figure 16.

Next, we consider

(34) $$N\left(t\right)=\sum_{d=1}^{25}R_{0}\left(d\right)N\left(t-d\right),\forall t\geq t_{0}+25,$$

and accordingly to the formula (17) and (20), with the initial condition for $t=t_{0},t_{0}+1,\ldots ,t_{0}+25$, we have

(35) $$N\left(t\right)=A_{1}\ln\left(\lambda_{1}\right)\lambda_{1}^{t}+e^{\alpha_{2}t}\left[{\hat{A}}_{2}\cos\left(\omega_{2}t\right)+{\hat{B}}_{2}\sin\left(\omega_{2}t\right)\right]+e^{\alpha_{3}t}\left[{\hat{A}}_{3}\cos\left(\omega_{3}t\right)+{\hat{B}}_{3}\sin\left(\omega_{3}t\right)\right],$$

with

(36) $${\hat{A}}_{2}=\alpha_{2}A_{2}+\omega_{2}B_{2},{\hat{B}}_{2}=-\omega_{2}A_{2}+\alpha_{2}B_{2},{\hat{A}}_{3}=\alpha_{3}A_{3}+\omega_{3}B_{3}\;\mathrm{and}\;{\hat{B}}_{3}=-\omega_{3}A_{3}+\alpha_{3}B_{3}.$$

In (24)–(26) we use the parameter values estimated in Section 3.1.

**Figure 19:**
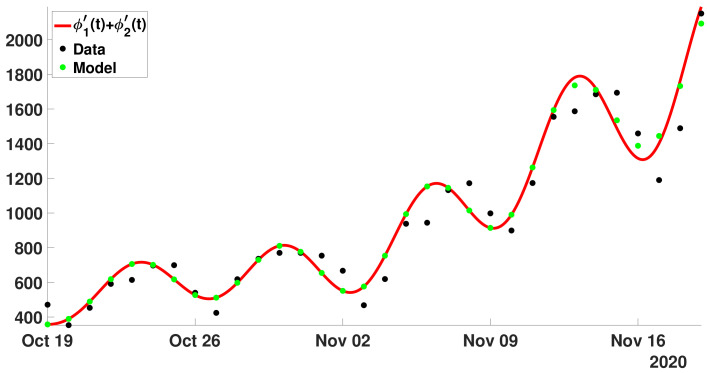
In this figure, we plot the daily number of reported cases data between 19 October and 19 November 2020 (black dots). The red curve corresponds to $\phi_{1}^{\prime }+\phi_{2}^{\prime }$, and the green dots correspond (34) and (35) whenever $R_{0}\left(d\right)$ comes from the average distribution (i.e., the red curve in Figure 15). We observe a very good match between the green dots and the red curve (the phenomenological model).

In Figure 19, we see the mean distribution $d\mapsto R_{0}\left(d\right)$ permits to produce oscillations around the tendency for the daily number of cases. It is important to note that without the third peak in Figure 16 we do not obtain such a good correspondence between the model and the data.

## 4. Discussion

In this article, we start by investigating the connection between a signal decomposed into a sum of damped or amplified oscillations and a renewal equation. Namely, we connect the daily number of reported cases written as

$$N\left(t\right)=N_{1}e^{\alpha_{1}t}\left[\cos\left(\omega_{1}t\right)+i\sin\left(\omega_{1}t\right)\right]+\ldots +N_{n}e^{\alpha_{n}t}\left[\cos\left(\omega_{n}t\right)+i\sin\left(\omega_{n}t\right)\right],\;\forall t\geq t_{1}-n,$$

with the renewal equation

$$N\left(t\right)=\sum_{d=1}^{n}R_{0}\left(d\right)N\left(t-d\right),\forall t\geq t_{1}.$$

In the context of epidemic time series, a spectral method usually refers to the Fourier decomposition of a periodic signal. In the present paper, the data are not periodic and are composed of an exponential function (Malthusian growth) perturbed with some damped oscillating functions. So we use complex numbers with non-null real parts. We refer to Cazelles et al. [33] for more results about time series.

### 4.1. Data over Ten Days

We can notice in Figure 9 and Figure 10 and Table 1 that the daily reproduction number as well as the instantaneous reproduction number are estimated. Concerning the instantaneous (or effective) reproduction number $R_{e}\left(t\right)$ [43,44] estimated by [42], which equals 1.1 at the 19th of October 2020, the best fit corresponds to n=7 days (see (c) in Figure 9). This value of the duration of the contagiousness period is close to the values 6 or 7 days and are close to the values estimated from the virulence measured in [14,45,46]. In Figure 10, we always obtain a U-shaped distribution for the curve of daily reproduction numbers. This corresponds to the biphasic form of the virulence already observed in respiratory viruses, such as influenza, as recalled in the Introduction.

This temporal behavior of the contagiousness can correspond to the evolution of contagious symptoms like cough or spitting, which diminish during the innate immune response, followed by a comeback of the symptoms before the adaptive immune response (whenever the innate defense has been overcome by the virus). If the innate cellular immunity has been not sufficient for eliminating the virus, the viral load again increases, causing a reappearance of the symptoms before the adaptive immunity (cellular and humoral) occurs, which results in a transient decrease in contagiousness between the two immunologic phases. The medical recommendations are, in the case of U-shaped contagiousness, never to take a transient improvement for a permanent disappearance of the symptoms and to stay at home to avoid a bacterial secondary infection that is possibly fatal.

The estimation of the daily reproduction numbers in the COVID-19 outbreak constitutes an important issue. At the public health level, to publish only the sum of the daily reproduction numbers, that is, to say the basic reproduction number $R_{0}$ or the effective reproduction number Re, could suffice for controlling and managing the behavior of a whole population with mitigation or vaccination measures. At the individual level, it is important to know the existence of a minimum of the daily reproduction numbers, which generally corresponds to a temporary clinical improvement, after a partial success of the innate immune defense. This makes it possible to advise the patient to continue to respect his own isolation, prevention, and therapy choices (depending on his vaccination state) even if this transient clinical improvement has occurred. The present methodology allows also to estimate both the individual contagiousness duration in a dedicated age class and also its seasonal variations, which is crucial for optimizing the benefit–risk decisions of the public and individual health policies.

### 4.2. Data over One Month

Over one month, we obtain a daily reproduction number with three peaks. Each peak is centered respectively on 7 days, 14 days, and 21 days. These quantities coincide with the period of 7 days and 21 days obtained in Figure 14 in fitting the first residual when we subtract the exponential growth first fit to the cumulative data. As far as we understand the problem, that is the period of 21 days in the data, which induces the third peak. This third peak is very suspicious. Nevertheless, the data lead us to such a shape for the daily reproductive number. We also tried to run Figure 19 without the third peak, and we obtained a bad fit to the data, while with this third peak, the fit is good. One may also note that the 21-day period is insignificant for the ACF and the PACF in Figure 12.

Several possibilities exist to explain this strange shape for the daily reproduction number using the data over one month. One possible explanation is that the Japanese population should be subdivided into several groups having very different infection dynamics (at the level of a single patient). Here we have in mind the patient with a short infection period but high transmissibility (super spreaders) versus the patient with a long infection period with mild symptoms.

We suspect that such a shape for the daily reproduction number could be attributed to the time since infection to report a case. The daily number of reported cases would be obtained from N(t), and the daily number of new infected cases by using the following model

$$D\left(t\right)=f\sum_{d=1}^{q}K\left(d\right)N\left(t-d\right),$$

where the integer q≥1 is the maximum number of days needed to report a case, f∈[0,1] is the fraction reported, and K(d)≥0 is the probability to report a case after *d* days. Therefore, we must have

$$\sum_{d=1}^{q}K\left(d\right)=1.$$

### 4.3. Perspectives and Conclusions

In the present paper, we only consider the Japanese data in the exponential phase of the third epidemic wave.

The case of Japan seems emblematic to us, as it corresponds to a wave of well-identified new cases following a clearly characterized endemic phase. The exponential growth phenomenon being transitory, this explains the relatively limited duration of the sampling, which corresponds to a period in days during which the epidemiological parameters (such as the transmission rate) can be considered as constant. It is in such circumstances where the Gaussian nature of the errors is difficult to prove, due to the small sampling, such that similar methods based on wavelets have been proposed (Walden and Hosken [37]).

The method of the present paper should be applied to several countries for each epidemic wave to obtain a more systematic study. For the moment, over one month, we obtained a shape for the daily reproduction number that follows the data very well. However, we are suspicious about the third peak. We suspect that the default of our analysis is coming from the model itself. Such a question has been recently studied by Ioannidis and his collaborators in [47], and we believe that we are facing such modeling difficulties.

## Figures and Tables

**Table 1:** The above reproduction numbers are obtained by using the formula $R_{0}=\sum_{d=1}^{n}R_{0}\left(d\right)$.

n	3	5	6	7
$R_{0}$	1.02	1.04	1.06	1.07

**Figure A1:**
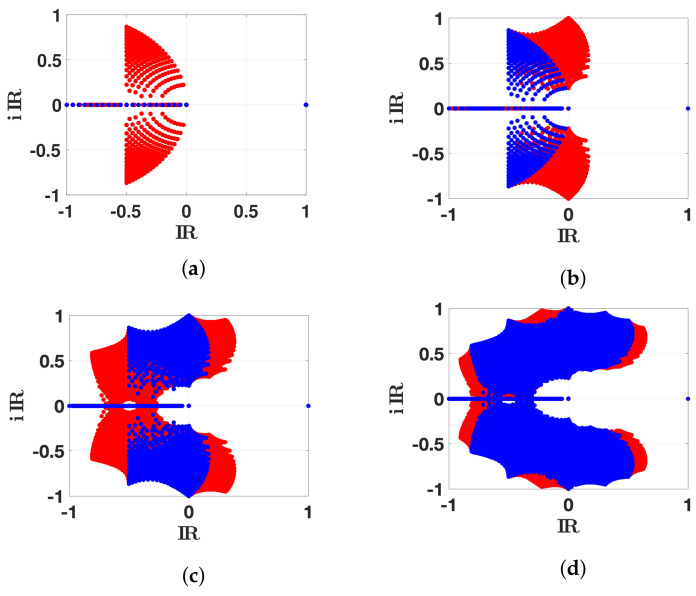
We plot all the spectrum’s locations for Markovian Leslie matrices on a mesh whenever n=3 in (**a**), n=4 in (**b**), n=5 in (**c**), and n=7 in (**d**). Here the dominant eigenvalue is always 1, and we can see the corresponding isolated blue dot. The blue region corresponds to the spectrum of Markovian Leslie matrices whenever $R_{0}\left(n\right)=0$. The red region corresponds to the spectrum of Markovian Leslie matrices whenever $R_{0}\left(n\right)>0$.

**Figure A2:**
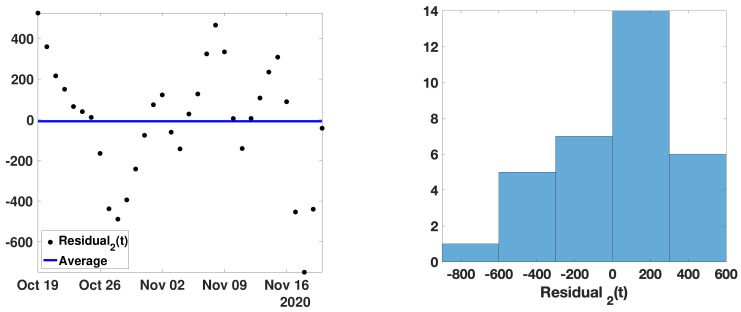
In this figure, we plot ${\mathrm{Residual}}_{2}\left(t\right)$.

## Data Availability

No data were produced for this study.

## Appendix A. Non Identifiability Result

From Formula (13), we deduce that the characteristic (12) has exactly one positive solution. By the Perron–Frobenius theorem applied to the Leslie matrix *L* defined by (11), we know that (by considering the norm of linear operator) the spectral radius of *L*

$$r\left(L\right):=\lim_{n\mapsto +\infty }{∥L^{n}∥}_{L(R)}^{1/n}>0,$$

is the unique positive solution of (12). Moreover, all the remaining eigenvalues have a modulus smaller or equal to r(L). We refer to ([48], Chapter 4), for more results about this subject.

**Non identifiability result:** Let $\lambda_{🟉}>0$ and $N_{🟉}\neq 0$. Then

$$N\left(t\right)=N_{🟉}\lambda_{🟉}^{t-t_{1}},\forall t\geq t_{1},$$

is a known solution of (8) if and only if $\lambda_{🟉}$ is a solution of the characteristic equation.

Assume that $d\in \left[1,n\right]\mapsto R^{🟉}\left(d\right)\geq 0$ is given, and satisfies

$$\sum_{d=1}^{n}R^{🟉}\left(d\right)>0.$$

Then if we define

$$R_{0}\left(a\right)=\frac{R^{🟉}\left(a\right)}{\sum_{d=1}^{n}R^{🟉}\left(d\right)\lambda_{🟉}^{-d}},\forall a=1,\ldots ,n,$$

we deduce that the equation (12) is satisfied for $\lambda =\lambda_{🟉}$, and $N\left(t\right)=N_{🟉}\lambda_{🟉}^{t-t_{1}}$ is a solution of (8). We conclude that a single function $N\left(t\right)=N_{🟉}\lambda_{🟉}^{t-t_{1}}$ is not enough to identify $R_{0}\left(1\right),R_{0}\left(2\right),R_{0}\left(3\right),\ldots ,R_{0}\left(n\right)$.

## Appendix B. Identifiability Result

**Assumption** **A1.**
*Assume that* $\lambda_{1},\ldots ,\lambda_{n}\in C$ *are nonzero complex numbers, and are separated two by two. That is,*

$$\lambda_{i}\neq 0,\forall i=1,\ldots ,n.$$

*and*

$$\lambda_{i}\neq \lambda_{j},\;whenever\;i\neq j.$$

**Remark** **A1.**
*Since the coefficients of the characteristic Equation (12) are all real, we could also impose that the conjugate of each eigenvalue belongs to the spectrum. That is*

$${\bar{\lambda }}_{i}\in \left\{\lambda_{1},\ldots ,\lambda_{n}\right\},\forall i=1,\ldots ,n.$$

*However, that is not necessary in this subsection.*

**Remark** **A2.**
*When all the eigenvalues are real, the above assumption will be satisfied if and only if* $\lambda_{1},\ldots ,\lambda_{n}\in R$ *are nonzero real numbers which are pairwise distinct. Up to a permutation, that is*

$$\lambda_{i}\neq 0,\forall i=1,\ldots ,n,$$

*and*

$$\lambda_{1}<\lambda_{2}<\ldots <\lambda_{n}.$$

**Lemma** **A1.**
*Let Assumption A1 be satisfied. Assume that each* $\lambda_{i}\in C$ *satisfies the characteristic Equation (12). Then the Leslie matrix L defined by (11) is diagonalizable (and invertible); moreover, for each* $U_{1},U_{2},\ldots ,U_{n}\in C$ *,*

$$U\left(t\right)=U_{1}\lambda_{1}^{t}+U_{2}\lambda_{2}^{t}+\ldots +U_{n}\lambda_{n}^{t},\;\forall t\geq t_{1}-n,$$

*is a solution of (8). That is to say,*

$$U\left(t\right)=\sum_{d=1}^{n}R_{0}\left(d\right)U\left(t-d\right),\forall t\geq t_{1}.$$

**Identification of the components $U_{i}$ from the values of t↦N(t):** Assume that the values of N(t) are given for $t=t_{1},\ldots ,t_{1}+n-1$. We claim that we can compute $U_{1},U_{2},U_{3},\ldots ,U_{n}\in C$. Indeed,

$$\begin{array}{cccc} N(t_{1}) & =U_{1}\lambda_{1}^{t_{1}} & +U_{2}\lambda_{2}^{t_{1}} & +\ldots +U_{n}\lambda_{n}^{t_{1}},\;\\ N(t_{1}+1) & =U_{1}\lambda_{1}^{t_{1}+1} & +U_{2}\lambda_{2}^{t_{1}+1} & +\ldots +U_{n}\lambda_{n}^{t_{1}+1},\;\\ \; & \vdots \\ N(t_{1}+n-1) & =U_{1}\lambda_{1}^{t_{1}+n-1} & +U_{2}\lambda_{2}^{t_{1}+n-1} & +\ldots +U_{n}\lambda_{n}^{t_{1}+n-1}, \end{array}$$

can be rewritten as the system

(A1) $$(\begin{array}{c} N(t_{1})\\ N(t_{1}+1)\\ \vdots \\ N(t_{1}+n-1) \end{array})=(\begin{array}{cccc} \lambda_{1}^{t_{1}} & \lambda_{2}^{t_{1}}\ldots \ldots \ldots \ldots & \lambda_{n}^{t_{1}}\\ \lambda_{1}^{t_{1}+1} & \lambda_{2}^{t_{1}+1}\ldots \ldots \ldots & \lambda_{n}^{t_{1}+1}\\ \vdots & \vdots \;\;\; & \vdots & \\ \lambda_{1}^{t_{1}+n-1} & \lambda_{2}^{t_{1}+n-1}\ldots \ldots & \lambda_{n}^{t_{1}+n-1} \end{array})(\begin{array}{c} U_{1}\\ U_{2}\\ \vdots \\ U_{n} \end{array}).$$

The determinant of the above Vandermonde-like matrix

$$\det(\begin{array}{cccc} \lambda_{1}^{t_{1}} & \lambda_{2}^{t_{1}}\ldots \ldots \ldots \ldots & \lambda_{n}^{t_{1}}\\ \lambda_{1}^{t_{1}+1} & \lambda_{2}^{t_{1}+1}\ldots \ldots \ldots & \lambda_{n}^{t_{1}+1}\\ \vdots & \vdots \;\;\; & \vdots & \\ \lambda_{1}^{t_{1}+n-1} & \lambda_{2}^{t_{1}+n-1}\ldots \ldots & \lambda_{n}^{t_{1}+n-1} \end{array})$$

$$=\lambda_{1}^{t_{1}}\lambda_{2}^{t_{2}}\ldots \lambda_{n}^{t_{n}}\det(\begin{array}{cccc} 1 & 1\ldots \ldots \ldots \ldots & 1\\ \lambda_{1}^{-1} & \lambda_{2}^{-1}\ldots \ldots \ldots & \lambda_{n}^{-1}\\ \vdots & \vdots \;\;\; & \vdots & \\ \lambda_{1}^{-(n-1)} & \lambda_{2}^{-(n-1)}\ldots \ldots & \lambda_{n}^{-(n-1)} \end{array}),$$

therefore,

$$\det(\begin{array}{cccc} \lambda_{1}^{t_{1}} & \lambda_{2}^{t_{1}}\ldots \ldots \ldots \ldots & \lambda_{n}^{t_{1}}\\ \lambda_{1}^{t_{1}+1} & \lambda_{2}^{t_{1}+1}\ldots \ldots \ldots & \lambda_{n}^{t_{1}+1}\\ \vdots & \vdots \;\;\; & \vdots & \\ \lambda_{1}^{t_{1}+n-1} & \lambda_{2}^{t_{1}+n-1}\ldots \ldots & \lambda_{n}^{t_{1}+n-1} \end{array})=\lambda_{1}^{-1}\lambda_{2}^{-1}\ldots \lambda_{n}^{-1}\prod_{1\leq i<j\leq n}\left(\lambda_{i}^{-1}-\lambda_{j}^{-1}\right).$$

Therefore, under Assumption A1, this determinant is non null, and we obtain the following result.

**Proposition** **A1.** *Let Assumption A1 be satisfied. Then we can compute the components*$U_{1},\ldots ,U_{n}$*in function of the given elements of the trajectory*$N\left(t_{1}\right),\ldots ,N\left(t_{1}+n-1\right)$*by solving the linear system *(A1)*, and*

$$(\begin{array}{c} U_{1}\\ U_{2}\\ \vdots \\ U_{n} \end{array})={\left(\begin{array}{cccc} \lambda_{1}^{t_{1}} & \lambda_{2}^{t_{1}}\ldots \ldots \ldots \ldots & \lambda_{n}^{t_{1}}\\ \lambda_{1}^{t_{1}+1} & \lambda_{2}^{t_{1}+1}\ldots \ldots \ldots & \lambda_{n}^{t_{1}+1}\\ \vdots & \vdots \;\;\; & \vdots & \\ \lambda_{1}^{t_{1}+n-1} & \lambda_{2}^{t_{1}+n-1}\ldots \ldots & \lambda_{n}^{t_{1}+n-1} \end{array}\right)}^{-1}(\begin{array}{c} N(t_{1})\\ N(t_{1}+1)\\ \vdots \\ N(t_{1}+n-1) \end{array}).$$

**Identification of the component $R_{0}\left(d\right)$ from the $\lambda_{i}$:** By assuming that each $\lambda_{i}$ is a solution of the characteristic Equation (12), we obtain

(A2) $$\begin{array}{cc} 1 & =R_{0}\left(1\right)\lambda_{1}^{-1}+R_{0}\left(2\right)\lambda_{1}^{-2}+\ldots +R_{0}\left(n\right)\lambda_{1}^{-n},\;\\ 1 & =R_{0}\left(1\right)\lambda_{2}^{-1}+R_{0}\left(2\right)\lambda_{2}^{-2}+\ldots +R_{0}\left(n\right)\lambda_{2}^{-n},\;\\ \; & \vdots \\ 1 & =R_{0}\left(1\right)\lambda_{n}^{-1}+R_{0}\left(2\right)\lambda_{n}^{-2}+\ldots +R_{0}\left(n\right)\lambda_{n}^{-n}, \end{array}$$

which rewrites in the matrix form as

$$(\begin{array}{c} 1\\ 1\\ \vdots \\ 1 \end{array})=(\begin{array}{ccc} \lambda_{1}^{-1} & \lambda_{1}^{-2}\ldots \ldots & \lambda_{1}^{-n}\\ \lambda_{2}^{-1} & \lambda_{2}^{-2}\ldots \ldots & \lambda_{2}^{-n}\\ \vdots & \vdots \;\; & \vdots \\ \lambda_{n}^{-1} & \lambda_{n}^{-2}\ldots \ldots & \lambda_{n}^{-n} \end{array})(\begin{array}{c} R_{0}\left(1\right)\\ R_{0}\left(2\right)\\ \vdots \\ R_{0}\left(n\right) \end{array}).$$

Under Assumption A1 the Vandermonde-like matrix

$$\left(\begin{array}{ccc} \lambda_{1}^{-1} & \lambda_{1}^{-2}\ldots \ldots & \lambda_{1}^{-n}\\ \lambda_{2}^{-1} & \lambda_{2}^{-2}\ldots \ldots & \lambda_{2}^{-n}\\ \vdots & \vdots \;\; & \vdots \\ \lambda_{n}^{-1} & \lambda_{n}^{-2}\ldots \ldots & \lambda_{n}^{-n} \end{array}\right)$$

is invertible, because

$$\det(\begin{array}{ccc} \lambda_{1}^{-1} & \lambda_{1}^{-2}\ldots \ldots & \lambda_{1}^{-n}\\ \lambda_{2}^{-1} & \lambda_{2}^{-2}\ldots \ldots & \lambda_{2}^{-n}\\ \vdots & \vdots \;\; & \vdots \\ \lambda_{n}^{-1} & \lambda_{n}^{-2}\ldots \ldots & \lambda_{n}^{-n} \end{array})=\lambda_{1}^{-1}\lambda_{2}^{-1}\ldots \lambda_{n}^{-1}\det(\begin{array}{ccc} 1 & \lambda_{1}^{-1}\ldots \ldots & \lambda_{1}^{-(n-1)}\\ 1 & \lambda_{2}^{-1}\ldots \ldots & \lambda_{2}^{-(n-1)}\\ \vdots & \vdots \;\; & \vdots \\ 1 & \lambda_{n}^{-1}\ldots \ldots & \lambda_{n}^{-(n-1)} \end{array})$$

hence

$$\det(\begin{array}{ccc} \lambda_{1}^{-1} & \lambda_{1}^{-2}\ldots \ldots & \lambda_{1}^{-n}\\ \lambda_{2}^{-1} & \lambda_{2}^{-2}\ldots \ldots & \lambda_{2}^{-n}\\ \vdots & \vdots \;\; & \vdots \\ \lambda_{n}^{-1} & \lambda_{n}^{-2}\ldots \ldots & \lambda_{n}^{-n} \end{array})=\lambda_{1}^{-1}\lambda_{2}^{-1}\ldots \lambda_{n}^{-1}\prod_{1\leq i<j\leq n}\left(\lambda_{i}^{-1}-\lambda_{j}^{-1}\right)\neq 0.$$

Therefore, we can compute the component of the map $d\in \left[1,n\right]\mapsto R_{0}\left(d\right)$ by solving a linear system involving the eigenvalues of the characteristic equation.

**Theorem** **A1**.
*Let Assumption A1 be satisfied. Then the following properties are equivalent*

(i) *The set*$\left\{\lambda_{1},\ldots ,\lambda_{n}\right\}$*is the spectrum of the Leslie matrix L defined in *(11)*.*
(ii) *Each element of*$\left\{\lambda_{1},\ldots ,\lambda_{n}\right\}$*satisfies *(A2)*.*
(iii) *The elements* $\left\{\lambda_{1},\ldots ,\lambda_{n}\right\}$ *satisfy*
  (A3)
  $${\left(\begin{array}{ccc} \lambda_{1}^{-1} & \lambda_{1}^{-2}\ldots \ldots & \lambda_{1}^{-n}\\ \lambda_{2}^{-1} & \lambda_{2}^{-2}\ldots \ldots & \lambda_{2}^{-n}\\ \vdots & \vdots \;\; & \vdots \\ \lambda_{n}^{-1} & \lambda_{n}^{-2}\ldots \ldots & \lambda_{n}^{-n} \end{array}\right)}^{-1}(\begin{array}{c} 1\\ 1\\ \vdots \\ 1 \end{array})=(\begin{array}{c} R_{0}\left(1\right)\\ R_{0}\left(2\right)\\ \vdots \\ R_{0}\left(n\right) \end{array}).$$

In Figure A1, we plot all the spectrum’s location for Markovian Leslie matrices on a mesh. We can observe the changes of location of the spectrum depending of the dimension *n*. It seems that the spectrum is fielding more and more the unit circle in C when the dimension increases. We refer to Kirkland [49] for more results going in that direction.

**Continuous dependency of the component $R_{0}\left(d\right)$ with respect to the $\lambda_{i}$:** Define the set $\Omega \subset C^{n}$ of all the elements $\Lambda =\left\{\lambda_{1}^{🟉},\ldots ,\lambda_{n}^{🟉}\right\}\in C^{n}$ satisfying Assumption A1. For each $\Lambda =\left\{\lambda_{1}^{🟉},\ldots ,\lambda_{n}^{🟉}\right\}\in \Omega ,$ we define

$$M\left(\Lambda \right)=(\begin{array}{ccc} \lambda_{1}^{-1} & \lambda_{1}^{-2}\ldots \ldots & \lambda_{1}^{-n}\\ \lambda_{2}^{-1} & \lambda_{2}^{-2}\ldots \ldots & \lambda_{2}^{-n}\\ \vdots & \vdots \;\; & \vdots \\ \lambda_{n}^{-1} & \lambda_{n}^{-2}\ldots \ldots & \lambda_{n}^{-n} \end{array}),\forall \Lambda =\left\{\lambda_{1},\ldots ,\lambda_{n}\right\}\in \Omega .$$

**Theorem** **A2.**
*Consider a sequence* ${\left\{\Lambda^{m}=\left\{\lambda_{1}^{m},\ldots ,\lambda_{n}^{m}\right\}\right\}}_{m\geq 0}\subset \Omega ,$ *and a point* $\Lambda^{🟉}=\left\{\lambda_{1}^{🟉},\ldots ,\lambda_{n}^{🟉}\right\}\in \Omega$ *(i.e., all satisfying Assumption A1). Assume that*

$$\lim_{m\mapsto +\infty }\Lambda^{m}=\Lambda^{🟉},$$

*then*

$$\lim_{m\mapsto +\infty }R_{0}^{m}=R_{0}^{🟉},$$

*where*

$$R_{0}^{m}=M{\left(\Lambda^{m}\right)}^{-1}(\begin{array}{c} 1\\ 1\\ \vdots \\ 1 \end{array}),\forall m\in N,\;\mathrm{and}\;R_{0}^{🟉}=M{\left(\Lambda^{🟉}\right)}^{-1}(\begin{array}{c} 1\\ 1\\ \vdots \\ 1 \end{array}).$$

**Proof.** We have

$$(\begin{array}{c} 1\\ 1\\ \vdots \\ 1 \end{array})=M\left(\Lambda^{m}\right)R_{0}^{m},\forall n\in N,\;\mathrm{and}\;(\begin{array}{c} 1\\ 1\\ \vdots \\ 1 \end{array})=M\left(\Lambda^{🟉}\right)R_{0}^{🟉}.$$

Subtracting the two above quantities, we obtain

(A4) $$0=M\left(\Lambda^{m}\right)R_{0}^{m}-M\left(\Lambda^{🟉}\right)R_{0}^{🟉},$$

which is also equivalent to

$$0=M\left(\Lambda^{m}\right)R_{0}^{m}-M\left(\Lambda^{🟉}\right)\left[R_{0}^{🟉}-R_{0}^{m}\right]-M\left(\Lambda^{🟉}\right)R_{0}^{m},$$

hence,

$$R_{0}^{🟉}-R_{0}^{m}=M{\left(\Lambda^{🟉}\right)}^{-1}\left[M\left(\Lambda^{m}\right)-M\left(\Lambda^{🟉}\right)\right]R_{0}^{m}.$$

Setting

$$L_{m}=M{\left(\Lambda^{🟉}\right)}^{-1}\left[M\left(\Lambda^{m}\right)-M\left(\Lambda^{🟉}\right)\right]$$

and so

$$\lim_{m\mapsto +\infty }L_{m}=0_{M_{n}\left(C\right)}.$$

Now since

$$R_{0}^{🟉}-R_{0}^{m}=L_{m}R_{0}^{🟉}-L_{m}\left[R_{0}^{🟉}-R_{0}^{m}\right],$$

we deduce that

$$∥R_{0}^{🟉}-R_{0}^{m}∥\leq ∥L_{m}{∥}_{L(C^{n})}∥R_{0}^{🟉}∥+∥L_{m}{∥}_{L(C^{n})}∥R_{0}^{🟉}-R_{0}^{m}∥,$$

hence, for all m≥1 large enough (i.e., satisfying $∥L_{m}{∥}_{L(C^{n})}<1$)

$$∥R_{0}^{🟉}-R_{0}^{m}∥\leq \frac{∥L_{m}{∥}_{L(C^{n})}}{1-∥L_{m}{∥}_{L(C^{n})}}∥R_{0}^{🟉}∥,$$

and the proof is completed. □

## Appendix C. Identification of the Phenomenological Model

Here we assume that the daily number of reported cases has the following form

(A5) $$N\left(t\right)=N_{1}e^{\lambda_{1}t}+N_{2}e^{\lambda_{2}t}+N_{3}e^{\lambda_{3}t}+\ldots +N_{m}e^{\lambda_{m}t},$$

where $N_{1},\ldots ,N_{n}\in C$ are non null, and $\lambda_{1},\ldots ,\lambda_{n}\in C$ are pairwise distinct.

If we assume to know t↦N(t) for all positive integer values t=0,1,2,…, then we can compute the discrete Laplace transform

$$L\left(N\right)\left(\lambda \right)=\sum_{t=0}^{\infty }e^{-\lambda t}N\left(t\right),$$

which is well defined for all λ∈C such that

Reλ>maxi=1,…,nReλi.

By using (A5), we obtain

$$L\left(N\right)\left(\lambda \right)=\sum_{p=1}^{m}\frac{N_{p}}{1-e^{\lambda_{p}-\lambda }},$$

whenever Reλ>maxi=1,…,nReλi.

Let $k\in \left\{1,\ldots ,m\right\}$ be an integer such that

Reλk=maxi=1,…,nReλi,

we obtain

limλ↦λkReλ>Reλk|LNλ|=+∞.

The Laplace transform could be used to identify the unknown parameters $\lambda_{1},\ldots ,\lambda_{m}$ in (A5). Then by combining this idea with linear regression of $t\mapsto e^{\lambda_{k}t}$, we could identify the parameters $N_{k}$, then step by step compute all the parameters of N(t) in (A5).

In practice, we only know t↦N(t) on a finite time interval t=0,1,2,…,L. In that case, we can define the truncated Laplace transform as

$$L\left(N\right)\left(\lambda \right)=\sum_{t=0}^{L}e^{-\lambda t}N\left(t\right)$$

and we have by (27)

$$L\left(N\right)\left(\lambda \right)=\sum_{p=1}^{m}N_{p}\frac{1-e^{\left(\lambda_{p}-\lambda \right)\left(L+1\right)}}{1-e^{\left(\lambda_{p}-\lambda \right)}}.$$

The Laplace transform does not permit to detect the eigenvalues $\lambda_{k}$ (we tested without success some examples with values of complex numbers coming from the present article). Identification of the eigenvalues $\lambda_{k}$, whenever t↦N(t) is known only on a finite time interval, seems to be an open intriguing question.

## Appendix D. About Residual 2 (t) in Section 3.3

In Figure A2, we observe that average of ${\mathrm{Residual}}_{2}\left(t\right)={\mathrm{Residual}}_{1}\left(t\right)-\phi_{2}\left(t\right)$ is close to 0, but its histogram does not have the shape of a normal distribution. So, there might be some residual information in ${\mathrm{Residual}}_{2}\left(t\right)$.
